# Solute carrier family 12 member 5 promotes tumor invasion/metastasis of bladder urothelial carcinoma by enhancing NF-*κ*B/MMP-7 signaling pathway

**DOI:** 10.1038/cddis.2017.118

**Published:** 2017-03-23

**Authors:** J Y Liu, Y B Dai, X Li, K Cao, D Xie, Z T Tong, Z Long, H Xiao, M K Chen, Y L Ye, B Liu, J Tan, J Tang, Z Z Xu, Y Gan, Y H Zhou, F Deng, L Y He

**Affiliations:** 1 Department of Urology, The Third Xiangya Hospital of Central South University, Changsha 410013, China; 2 Institute of Prostate Disease of Central South University, Changsha 410013, China; 3 Center for Molecular Medicine, Xiangya Hospital, Central South University, Changsha 410008, China; 4 Department of Onology, The Third Xiangya Hospital of Central South University, Changsha 410013, China; 5 Department of Pathology, Sun Yat-sen University Cancer Center, Guangzhou 510060, China; 6 State Key Laboratory of Oncology in South China, Guangzhou 510060, China; 7 Department of Radiotherapy, The First Affiliated Hospital of Ahhui Medical University, Anhui 230022, China; 8 Department of Pathology, The Third Xiangya Hospital of Central South University, Changsha 410013, China; 9 Department of Urology, The Third Affiliated Hospital of Southern Medical University, Guangzhou 510630, China; 10 Department of Urology, Sun Yat-sen University Cancer Center, Guangdong 510060, China; 11 Department of Urology, Hunan Cancer Hospital, Changsha 410013, China

## Abstract

Solute carrier family 12 member 5 (SLC12A5), an integral membrane KCl cotransporter, which maintains chloride homeostasis in neurons, is aberrantly expressed and involved in the tumorigenesis of certain cancers. However, the clinical significance and biological role of SLC12A5 in human bladder urothelial carcinoma (BUC) remains unclear. In this study, the expression of SLC12A5 was examined in clinical specimens of primary BUC and in BUC cell lines using quantitative real-time reverse transcription polymerase chain reaction (qRT-PCR), western blot and immunohistochemistry (IHC). The prognostic value of SLC12A5 expression and its correlation with the clinicopathological features of patients with BUC were analyzed statistically. A series of *in vitro* and *in vivo* assays were performed to elucidate the effect of SLC12A5 in BUC and its underlying mechanisms. The present results showed that SLC12A5 expression was significantly increased in BUC tissues. SLC12A5 expression significantly correlated with the tumor node metastasis (TNM) stage. Kaplan–Meier survival curves showed that high SLC12A5 expression was associated with poor survival in patients with BUC. Multivariate analysis indicated that SLC12A5 expression was an independent prognostic marker for the survival of patients. Downregulation of SLC12A5 inhibited the migratory and invasive abilities of BUC cells *in vitro*, and knocking down SLC12A5 diminished BUC metastasis *in vivo*. Moreover, we identified that SLC12A5 promoted the migration and invasion of BUC by enhancing MMP-7 expression via NF-*κ*B-dependent transcription. Taken together, our findings indicated that SLC12A5 might function as a tumor metastasis promoting factor in the development and progression of BUC by regulating the NF-*κ*B/MMP-7 signaling pathway. Thus, SLC12A5 might be a prognostic marker as well as a therapeutic target for BUC.

Bladder urothelial carcinoma (BUC) is the fourth most common cancer in men, accounting for 7% of all cancer cases. In 2016, there will be 76 960 new cases of BUC in the USA alone, leading to 16 390 cancer-related deaths.^1^ Most patients with primary BUC initially present with non-muscle invasive carcinoma, whereas the remaining 20–25% of primary tumors are already muscle invasive at first diagnosis.^2, 3^ Radical cystectomy (RC) is the cornerstone of the management of invasive BUC and in superficial disease that is at high risk of recurrence and progression.^4^ Despite recent developments in therapeutic strategies including surgical resection and chemotherapy, the disease recurs in approximately 50% of patients, and the 5-year overall survival rate is only 6% for patients who develop or present with distant metastatic disease.^1^ Therefore, the high metastasis of BUC after RC surgery has always been the major obstacle to enhancing the survival rate in clinical treatment. According to current studies, BUC metastatic potential is linked frequently to altered gene expression profiles; therefore, identifying the molecular mechanisms of the genes closely related to the metastasis of BUC is central to improving prognosis in patients with BUC.

Solute carrier family 12 member 5 (SLC12A5) is a potassium chloride cotransporter 2, which belongs to the SLC12.^5^ SLC12A5 was identified initially as an integral membrane KCl cotransporter that maintains chloride homeostasis in neurons.^6, 7^ Recently, two studies showed that mutated SLC12A5 is highly expressed in colorectal cancer at the single-cell level, but exhibited low prevalence at the population level; they then identified that mutant SLC12A5 has a potential oncogenic effect in colorectal cancer.^8, 9^ Moreover, studies confirmed that overexpression of SLC12A5 has a pivotal oncogenic role in colorectal carcinogenesis by inhibiting apoptosis via mediating apoptosis inducing factor-dependent and Endonuclease G-dependent apoptotic signaling pathway and promoting metastasis by regulating key elements of the matrix architecture.^10^ Collectively, these studies suggest that SLC12A5 is an oncogene that contributes to the progression and development of colorectal cancer. Despite these extensive investigations in colorectal cancer, the expression, clinical significance and biological function of SLC12A5 in BUC remains poorly understood. Therefore, the present study was conducted to determine the molecular mechanisms underlying the expression of SLC12A5 and its clinical significance in human BUC.

## Results

### Expression of SLC12A5 in BUC tissue samples

**Figure 1:**
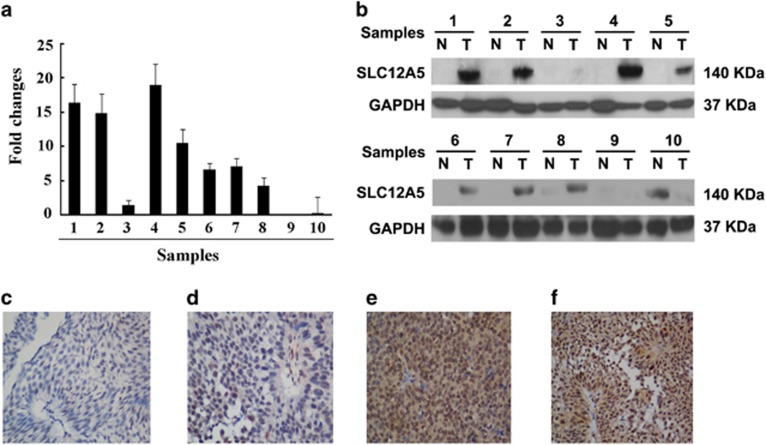
Expression of SLC12A5 mRNA and protein in human primary BUC surgical specimens as evaluated by qRT-PCR, western blot and IHC. (**a**) The mRNA expression of SLC12A5 is upregulated in 10 BUC tissues than in matched adjacent noncancerous tissues, analyzed by qRT-PCR analysis. (**b**) Representative results of expression of SLC12A5 protein in 10 paired BUC tissues and matched adjacent noncancerous tissues (T, BUC tissues; N, matched noncancerous tissues). (**c**–**f**) IHC analysis of SLC12A5 protein expression in primary BUC surgical specimens (× 400). (**c**) Low expression of SLC12A5 was observed in a BUC tissue (case 18), in which <20% of tumor cells demonstrated staining of SLC12A5. (**d**) Another BUC (case 89) tissue showed low expression of SLC12A5, in which 20–50% of tumor cells demonstrated staining of SLC12A5. (**e**) High expression of SLC12A5 was observed in a BUC case (case 8). (**f**) Strong SLC12A5 IHC signaling was detected in the BUC cells (case 26)

To investigate the expression status of SLC12A5 in BUC tissue, we conducted quantitative real-time reverse transcription polymerase chain reaction (qRT-PCR) and western blot analysis in 10 fresh paired samples from BUC patients. We found that SLC12A5 mRNA (Figure 1a) and protein (Figure 1b) expression were higher in human BUC tissues than in the paired adjacent nontumor tissues.

### Immunohistochemistry analysis of SLC12A5 expression in BUC clinical samples and its relationship to clinicopathological parameters

To identify the relationship between SLC12A5 expression and clinicopathological variables in human BUC, 148 paraffin-embedded primary BUC samples were used to examine SLC12A5 expression using immunohistochemistry (IHC) staining. In the SLC12A5-positive specimens, SLC12A5 was detected in the nucleus and cytoplasm in tumor cells (Figures 1c–e). Seventy-nine cases (53.4%) had high SLC12A5 levels; the remaining 69 cases (46.6%) had low SLC12A5 levels (Table 1). The correlations between SLC12A5 levels and various clinicopathological parameters are summarized in Table 1. Chi-square analysis revealed that the level of SLC12A5 in BUC tissues was only highly correlated with pN status (*P*=0.001) but not with age (*P*=0.975), gender (*P*=0.742), tumor size (*P*=0.781), tumor multiplicity (*P*=0.292), tumor grade (*P*=0.130) and pT status (*P*=0.079; Table 1).

### Relationship between SLC12A5 levels and survival

**Figure 2:**
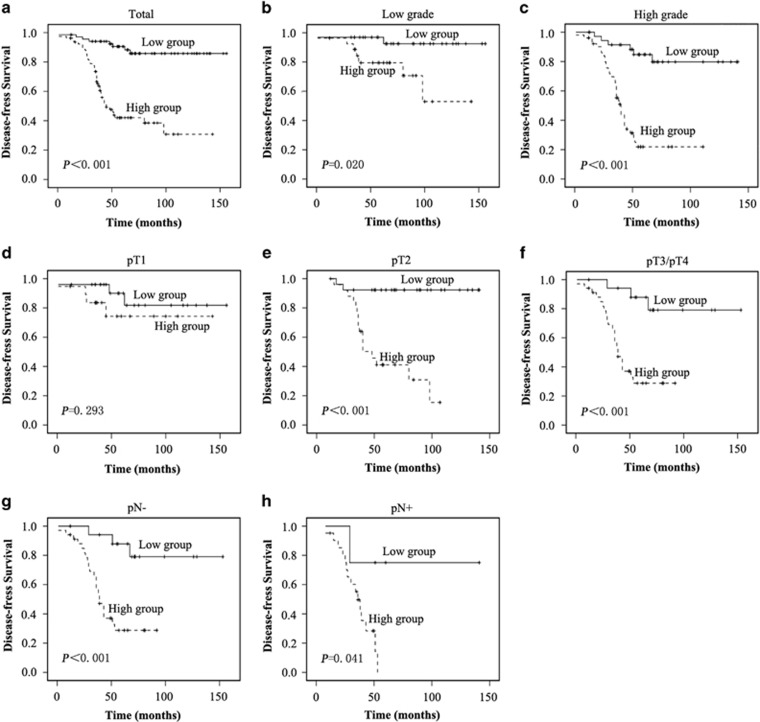
Kaplan–Meier survival curves for BUC patients after radical cystectomy according to SLC12A5 expression. (**a**) Significantly improved survival was observed in all BUC patients whose tumors exhibited low SLC12A5 expression *versus* those whose tumors exhibited high SLC12A5 expression (*P*<0.001). (**b**) Significantly improved survival was observed in low-grade BUC patients whose tumors exhibited low SLC12A5 expression *versus* those whose tumors exhibited high SLC12A5 expression (*P*=0.020). (**c**) Significantly improved survival was observed in high-grade BUC patients whose tumors exhibited low SLC12A5 expression *versus* those whose tumors exhibited high SLC12A5 expression (*P*<0.001). (**d**) Probability of survival of pT1 patients with BUC (*P*=0.293). (**e**) Significantly improved survival was observed in pT2 BUC patients whose tumors exhibited low SLC12A5 expression *versus* those whose tumors exhibited high SLC12A5 expression (*P*<0.001). (**f**) Significantly improved survival was observed in pT3/pT4 BUC patients whose tumors exhibited low SLC12A5 expression *versus* those whose tumors exhibited high SLC12A5 expression (*P*<0.001). (**g**) Significantly improved survival was observed in pN− BUC patients whose tumors exhibited low SLC12A5 expression *versus* those whose tumors exhibited high SLC12A5 expression (*P*<0.001). (**h**) Significantly improved survival was observed in pN+ BUC patients whose tumors exhibited low SLC12A5 expression *versus* those whose tumors exhibited high SLC12A5 expression (*P*<0.001)

The prognostic value of SLC12A5 for survival was evaluated by comparing high and low SLC12A5 levels in the patients with BUC. Log-rank testing and Kaplan–Meier analysis showed that higher SLC12A5 levels led to shorter survival, while lower SLC12A5 levels were associated with longer survival (*P*<0.001; Figure 2a). Moreover, patients with higher SLC12A5 levels survived for a significantly shorter time than those with low levels in both the low (*P*=0.020; Figure 2b) and high-grade subgroups (*P*<0.001; Figure 2c), in pT2 (*P*<0.001; Figure 2e), in pT3/pT4 (*P*<0.001; Figure 2f) stage, in pN− (*P*<0.001; Figure 2g) and in pN+ (*P*=0.041; Figure 2h).

These results indicated that a lower SLC12A5 level correlated significantly with improved prognosis in patients with BUC (Figure 2). The effect of SLC12A5 expression on patient survival was further evaluated by univariate and multivariate analyses. In the univariate analysis, low SLC12A5 levels were associated significantly with improved survival in patients with BUC (hazard ratio (HR), 6.945; 95% confidence interval (CI), 3.238–14.896; *P*<0.001; Supplementary Table 1). In multivariate Cox regression analysis, we found that the SLC12A5 level was also an independent prognostic marker for survival (HR, 2.323; 95% CI, 1.820–2.966; *P*<0.001; Supplementary Table 2). Our results indicated clearly that a high SLC12A5 level is associated with poor prognosis, suggesting that SLC12A5 might serve as a molecular prognostic marker for this aggressive disease.

### Downregulation of SLC12A5 inhibited migration and invasion of human BUC cells *in vitro*

**Figure 3:**
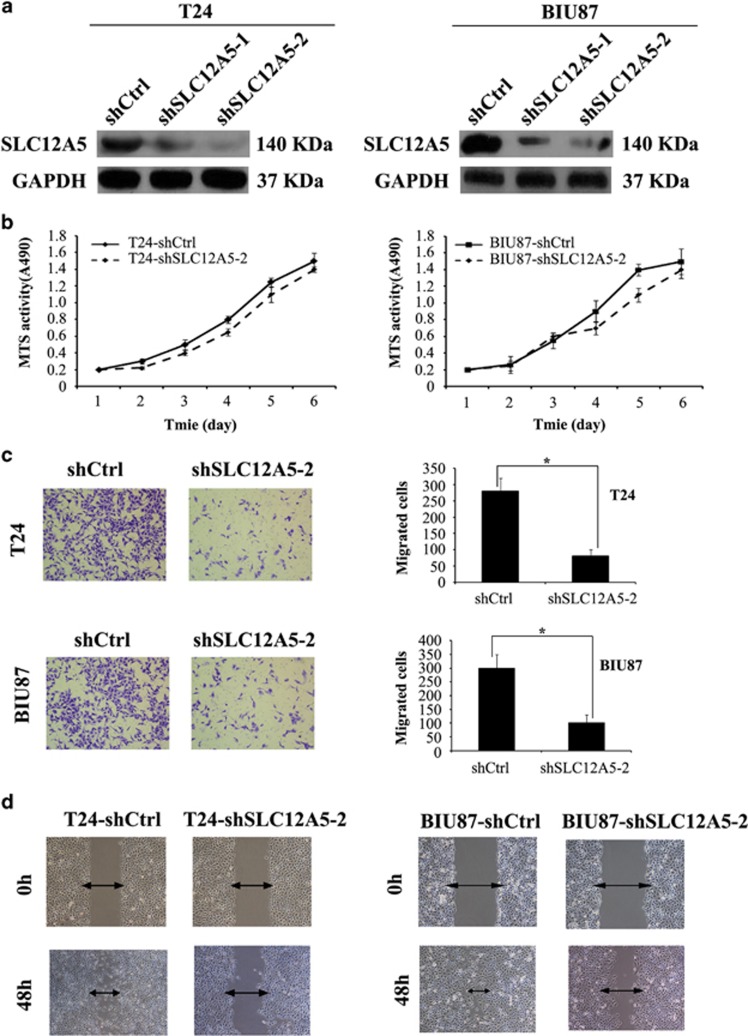

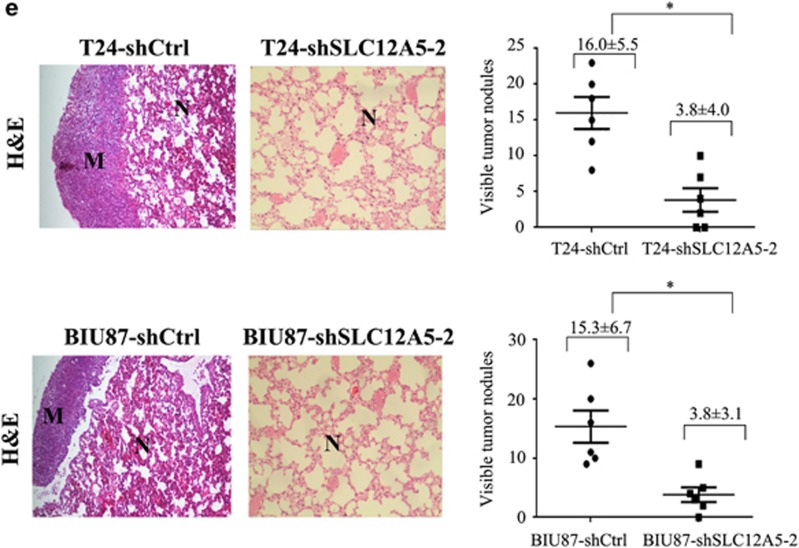
SLC12A5 enhances the migration and invasion of BUC cells *in vitro* and *in vivo*. (**a**) The expression of SLC12A5 was substantially decreased in SLC12A5-ShRNA BUC cells compared with that in ShCtrl BUC cells by western blot. (**b**) Suppression of SLC12A5 did not alter the cellular growth rate. (**c**) Representative micrographs and quantification of the invasiveness of SLC12A5-ShRNA BUC cells in the transwell matrix invasion assay compared with ShCtrl BUC cells. (**d**) Representative micrographs of the motility of SLC12A5-ShRNA BUC cells in the wound healing assay at 0 and 48 h compared with ShCtrl BUC cells. (**e**) Tail vein injection of BUC cells stably expressing SLC12A5 ShRNA or control shRNA into the nude mice led to metastasis to the lung. Left panel: image of a lung 2 months after tail vein injection of ShCtrl BUC cells and SLC12A5-ShRNA BUC cells. Right panel: number of metastases in lungs of mice (*n*=6 per group) 2 months after tail vein injection of ShCtrl BUC cells (mean±S.E.M., 16.0±5.5 for T24-ShCtrl cells; 15.3±6.7 for BIU-ShCtrl cells) and SLC12A5-ShRNA BUC cells (mean±S.E.M., 3.8±4.0 for T24-SLC12A5-ShRNA cells; 3.8±3.1 for BIU87-SLC12A5-ShRNA cells). The nodules were examined under an anatomical microscope; *P<0.01

To further identify the role of SLC12A5 in BUC progression, shRNAs were used to suppress endogenous SLC12A5 expression in two BUC cell lines, T24 and BIU87, which have high endogenous SLC12A5 expression. SLC12A5 downregulation at the protein level was confirmed via western blot (Figure 3a). Knocking down SLC12A5 did not alter the cellular growth rate *in vitro* (Figure 3b) and *in vivo* (Supplementary Figures 1A and B), suggesting that SLC12A5 does not have a role in controlling cellular growth. However, in the migration assay, we found that SLC12A5 knockdown in BUC cells suppressed the ability to migrate through transwell filter inserts significantly (Figure 3c). A similar result was obtained in the cell invasion assay: SLC12A5 knockdown suppressed the invasion ability of BUC cells (Figure 3d). These results suggested a pro-invasive role of SLC12A5 in human BUC T24 and BIU87 cell lines.

### SLC12A5 promotes BUC cells metastasis *in vivo*

To investigate the *in vivo* effect of SLC12A5 knockdown on metastasis, we used an experimental metastasis assay in which we injected T24 and BIU87 cells producing the control shRNA and SLC12A5 shRNA into the lateral tail vein of severe combined immunodeficiency (SCID) mice. In accordance with previous results, the mice injected with T24 and BIU87 cells expressing the SLC12A5 shRNA formed fewer nodes per lung compared with the mice injected with control shRNA-producing cells (3.8±4.0 *versus* 16.0±5.5, *P*=0.004; 3.8±3.1 *versus* 15.3±6.7, *P*=0.009 for T24 and BIU87 cells, respectively). Histological studies confirmed that the lesions were caused by extravasation and subsequent tumor growth of T24 and BIU87 cells into the lungs (Figure 3e). Our data indicated that SLC12A5 is involved in the control of BUC metastasis *in vivo*.

### SLC12A5 promotes human BUC cells' migration and invasion abilities by enhancing MMP-7 expression

**Figure 4:**
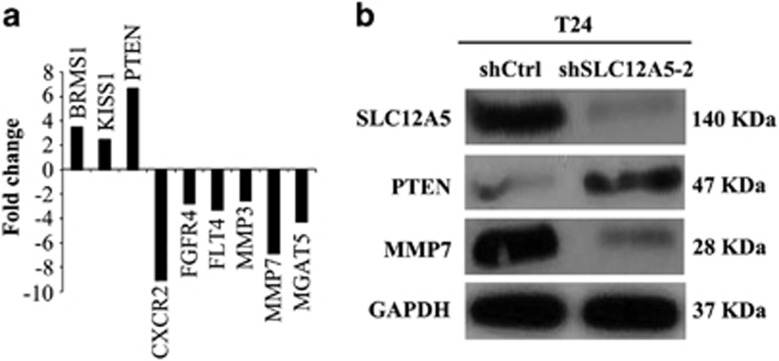
SLC12A5 regulated MMP-7 expression in BUC cells. (**a**) The nine genes, BRMS1, KISS1, PTEN, CXCR2, FGFR4, FLT4, MMP-3, MMP-7 and MAGT5, were examined >2-fold mRNA differential expression in SLC12A5-ShRNA -transfected T24 cells compared with that of T24-ShCtrl transfected by using a Human Tumor Metastasis RT^2^ Profiler PCR Array. (**b**) ShRNA-mediated SLC12A5 knockdown substantially upregulated PTEN expression and downregulated MMP-7 expression in T24 cells detected by western blot

To gain a further insight into the functions of SLC12A5 in BUC cell invasion and metastasis, the mRNA expression profiles of T24-SLC12A5-ShRNA cells were compared with T24-Control-ShRNA using a Human Tumor Metastasis RT^2^ Profiler PCR Array containing 84-cell metastasis-related genes. The results identified six upregulated and five downregulated genes (by >1.5-fold) in T24-SLC12A54-ShRNA cells compared with those in T24-Control-ShRNA cells (Supplementary Table 3). Subsequently, MMP-7, PTEN, BRMS1, KISS1, CXCR2, FGFR4, FLT4, MMP-3 and MAGT5, which exhibited >2-fold mRNA differences before and after SLC12A5 knockdown (Figure 4a), were selected and analyzed further by western blot. Consistent with that of mRNA expression in real-time PCR array, decreased protein expression of MMP-7 were demonstrated by western blot in T24 cells after SLC12A5 knockdown (Figure 4b). Utilizing the previous scoring criterions for IHC staining evaluation of MMP-7,^11^ positive expression of MMP-7 was observed in 92/148 (62.2%) of BUCs. In addition, a significant correlation between the levels of SLC12A5 and MMP-7 were evaluated in our BUC cohort, in which the frequency of cases with low SLC12A5 levels was significantly higher in negative MMP-7 expression cases (42/56 cases, 75.0%) compared with that in positive MMP-7 expression ones (27/92 cases, 29.3% *P*<0.001, Supplementary Table 4).

**Figure 5:**
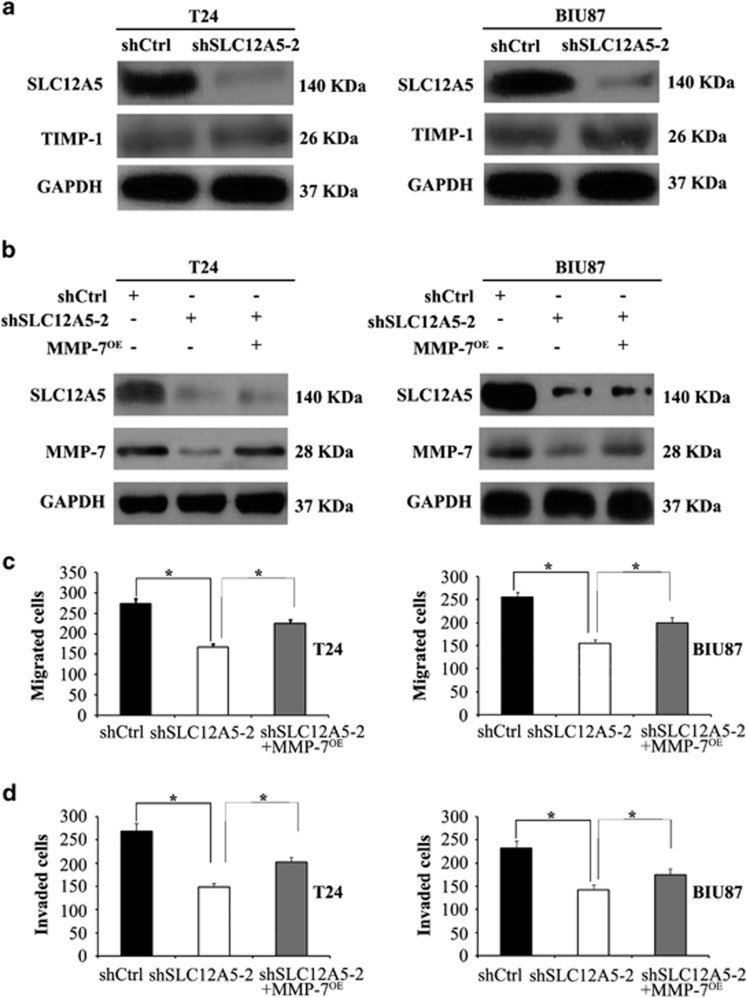
SLC12A5 promotes migration and invasion of BUC cells by enhancing MMP-7 expression. (**a**) Western blot of SLC12A5, MMP-7 and TIMP-1 from BUC cells transfected with the SLC12A5 ShRNA or control ShRNA. MMP-7 expression was downregulated independent of TIMP-1 in SLC12A5 knockdown BUC cells. (**b**) Western blot of SLC12A5 and MMP-7 from BUC cells transfected with the control ShRNA, SLC12A5 ShRNA or co-treated with overexpression of MMP-7 (MMP-7^OE^). (**c** and **d**) The reduction of migration and invasion regulated by SLC12A5 knockdown in BUC cells was rescued by overexpression of MMP-7. All the experiments were carried out in triplicate. Histograms represent means±S.D.; *P<0.01

Tissue inhibitors of matrix metalloproteinases (TIMPs) have the ability to inhibit the catalytic activity of matrix metalloproteinases (MMPs), and an imbalance between MMPs and TIMPs is responsible for cancer metastasis.^11^ TIMP-1 is the tissue inhibitor of MMP-7. To understand whether SLC12A5 regulates MMP-7 expression, we detected the expression of TIMP proteins. Unfortunately, data showed that TIMP-1 expression was unchanged when MMP-7 expression was downregulated in SLC12A5-knockdown T24 and BIU87 cells (Figure 5a). To determine whether altered MMP-7 expression has a role in SLC12A5 promotion of BUC migration and invasion, we first determined the protein levels of MMP-7 in T24 and BIU87 cells after SLC12A5 knockdown. The western blot results showed that the level of MMP-7 protein decreased in BUC cells after SLC12A5 knockdown (Figure 5b). Similarly, suppression of SLC12A5 decreased the activity of MMP-7 (Supplementary Figure 2). Moreover, MMP-7 overexpression led to rescued MMP-7 levels in SLC12A5 shRNA T24 and BIU87 cells (Figure 5b). As expected, MMP-7 overexpression rescued the ability of SLC12A5 shRNA to suppress T24 and BIU87 cells migration and invasion (Figures 5c and d). These data promoted us to investigate the potential mechanism of SLC12A5's regulation MMP-7 expression.

### SLC12A5 promotes MMP-7 expression via NF-*κ*B-dependent transcription

**Figure 6:**
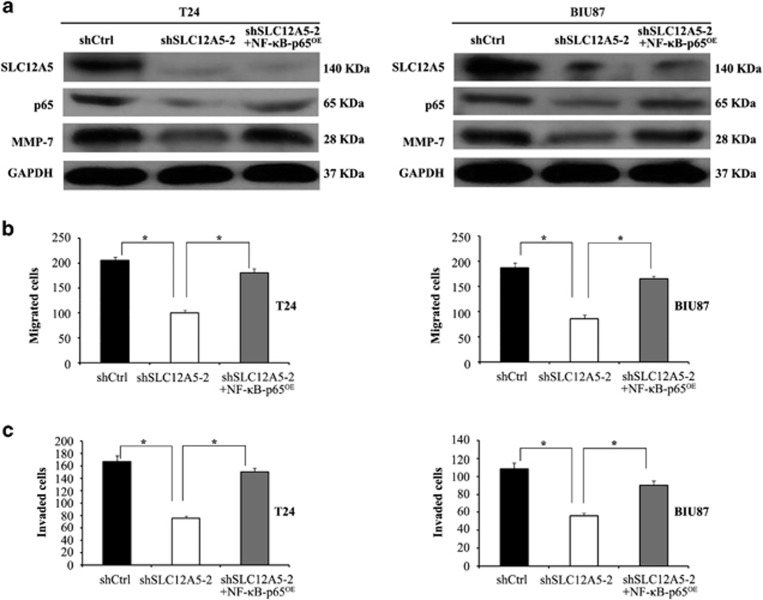
SLC12A5 promotes MMP-7 expression via NF-*κ*B-dependent transcription. (**a**) Western blot analysis of the relative protein levels of SLC12A5, MMP-7 and NF-*κ*B-p65 in three groups of ShCtrol, SLC12A5 ShRNA and SLC12A5 ShRNA co-treated with overexpression of NF-*κ*B-p65 (NF-*κ*B-p65^OE^) for both T24 and BIU87 stable cell lines. The overexpression of NF-*κ*B-p65 rescued the downregulation of MMP-7 expression induced by SLC12A5 knockdown. (**b** and **c**) The decreased ability of migration and invasion regulated by SLC12A5 knockdown was rescuced by overexpression of NF-*κ*B-p65 in both T24 and BIU87 stable cell lines. All experiments were carried out in triplicate. Histograms represent means±S.D.

Nuclear factor kappa B (NF-*κ*B) is a critical transcription factor activated in a large number of human cancers and has a crucial role in tumor development and progression.^12^ NF-*κ*B regulates downstream genes associated with metastasis and angiogenesis.^12, 13^ Its *κ*B site has been identified in the promoters of the genes that encode MMP-7,^13^ and the activation of NF-*κ*B induces membrane-type proteases (MT1-MMP), the activator of pro-MMP-7, which cleaves proteolytically pro-MMP-7 to generate functionally active MMP-7.^14^ Therefore, we hypothesized that SLC12A5 regulates MMP-7 expression via NF-*κ*B signaling pathway. To test this hypothesis, we first determined the protein levels of p65 (a subunit of NF-*κ*B) in T24 and BIU87 cells after SLC12A5 knockdown. The western blot results showed that the level of p65 protein decreased in BUC cells after SLC12A5 knockdown (Figure 6a). Furthermore, NF-*κ*B-p65 overexpression led to rescued MMP-7 protein in SLC12A5 shRNA T24 and BIU87 cells (Figure 6a); likewise, overexpression of NF-*κ*B-p65 rescued the downregulation of MMP-7 activity induced by SLC12A5 knockdown (Supplementary Figure 3). These data provide evidence that SLC12A5 modulates MMP-7 expression via the activity of the NF-*κ*B transcription factor.

To further confirm whether NF-*κ*B deactivation by SLC12A5 inhibition caused the downregulation of MMP-7 in human BUC cells, we also validated the mechanism by migration and invasion analysis. Migration and invasion abilities were decreased in T24-SLC12A5-ShRNA cells and BIU87-SLC12A5-ShRNA cells; however, these effects were rescued by overexpression of NF-*κ*B-p65 (Figures 6b and c). This result suggested that SLC12A5 might regulate migration and invasion via the NF-*κ*B/MMP-7 signaling pathway.

## Discussion

SLC12A5 is a functional gene located at human chromosome 20q13.12, which is one of the most frequently amplified regions in a variety of human malignancies.^15, 16, 17, 18^ Recently, SLC12A5 has been shown to have clinical/prognostic and functional significance in human colorectal and cervical cancer.^10, 19^ However, the expression and function of SLC12A5 in BUC, and its correlation with the clinicopathological features of patients with BUC, have not been investigated.

Thus, we sought to study its role in human BUC. We report, for the first time, an association between SLC12A5 expression and BUC progression. In the present study, a relatively large BUC cohort was studied. Our data showed upregulation of SLC12A5 levels in the majority of BUC tissues. Specifically, high SLC12A5 levels were associated with advanced disease progression, shorter survival and poor prognosis. Moreover, multivariate Cox proportional hazards regression analysis investigated that a high SLC12A5 level is a strong, independent negative prognostic indicator for BUC. Therefore, our results demonstrated an oncogenic role of SLC12A5 in BUC progression, and suggested that SLC12A5 is a potential novel therapeutic target in BUC patients.

Our clinical data prompted us to carry out a series of *in vitro* and *in vivo* experiments to explore the potential mechanisms. In the present study, using two human BUC cell lines, T24 and BIU87, the SLC12A5 protein was detectable by western blot in the cells, and the T24 and BIU87 cell lines showed high levels of endogenous SLC12A5. Further functional studies demonstrated that the inhibition of SLC12A5 expression by transfection with an shRNA in both T24 and BIU87 cells led to markedly reduced cell migration and invasion ability *in vitro*, but did not affect cell proliferation. In addition, in an experimental *in vivo* metastasis model using SCID mice, we further showed that the tail vein injection of SLC12A5-ShRNA T24 and BIU87 cells led to a significant decrease in the number of metastatic lesions in the lungs of mice, as compared with those in the lungs of mice injected with control T24 and BIU87 cells. Collectively, these data supported strongly the view that SLC12A5 has a crucial oncogenic role in the promotion of BUC cells invasive and/or metastatic process.

However, the molecular mechanisms by which SLC12A5 regulates BUC cell migration/invasion remain unclear. To gain a further insight into the downstream molecular events involving SLC12A5 and BUC invasiveness and/or metastasis, we used a cDNA microarray, qRT-PCR, western blot, and IHC. We revealed that the enhanced cell invasion and migration by SLC12A5 were mediated via downregulation of key anti-metastasis genes, including BRMS1, KISS1 and PTEN, and upregulation of important pro-metastasis genes MMP-7, CXCR2, FGFR4, FLT4, MMP-3 and MAGT5 (Supplementary Table 3). Subsequently, the protein levels of these nine genes were analyzed by western blot. Consistent with the mRNA expression in the qRT-PCR array, upregulated PTEN and downregulated MMP-7 in protein levels were observed after SLC12A5 knockdown in T24 cells. It appeared that in our BUC cells, SLC12A5 regulates cell migration/invasion via the regulation of PTEN and/or MMP-7 expression. To confirm these abservations in T24 cells, PTEN and MMP-7 levels were further examined by IHC in a relatively large cohort of BUCs. We found that 76.3% and 62.2% of carcinomas had negative expression of PTEN and positive expression of MMP-7 expression, respectively. Further correlation analysis demonstrated no significant correlation between the levels of SLC12A5 and PTEN. Interestingly, however, we observed a significant positive correlation between high levels of SLC12A5 and positive expression of MMP-7. Thus, it appears that in BUC cells, SLC12A5 regulates cell migration/invasion via the regulation of MMP-7 expression.

MMP-7 is one of the few MMPs that is produced directly by tumor cells, making it an attractive biomarker to identify an aggressive tumor phenotype.^20^ Elevated MMP-7 tissue levels correlate with poor patients survival in various cancers, such as colorectal, gastric, pancreatic and prostate cancer.^21, 22, 23^ In BUC, high MMP-7 gene expression proved to be an independent prognostic indicators of metastasis and lymph node metastasis.^24, 25, 26^ These observations, together with the results of our SLC12A5 functional studies in BUC cells, suggested that the upregulated expression of SLC12A5 in BUCs might involve the MMP-7 associated pathway and thus support cancer cell invasion and/or metastasis.

MMPs expression is controlled by specific, endogenous TIMPs and the imbalance between MMPs and TIMPs is responsible for cancer metastasis.^11^ TIMP-1 is the tissue inhibitor of MMP-7, whose negative regulation of MMP-7 is involved in several tumor metastasis processes.^27^ However, in our study, TIMP-1 did not seem to be the regulator of MMP-7 expression in BUC cells. These results prompted us to investigate the potential mechanism of how SLC12A5 regulates MMP-7 expression. Therefore, we turned to NF-*κ*B, another common regulator of MMP-7.

NF-*κ*B is a critical transcription factor that is activated in various types of human cancers and plays a crucial role in tumor development and progression.^12, 13^ NF-*κ*B signaling modulates several key biological processes during the development and progression of cancer by inducing the transcription of a variety of target genes that regulate cell proliferation, survival, invasion and angiogenesis.^28, 29^ NF-*κ*B is present constitutively in cells as a heterodimer, consisting of a p50 DNA-binding subunit and a p65 transactivating subunit.^30^ Accumulating evidence has demonstrated that the expressions of several MMPs (including MMP-7) are regulated by p65 subunit upregulation and nuclear translocation-induced NF-*κ*B activation in many human cancers.^13^ The present results showed that downregulation of SLC12A5 using an shRNA decreased the protein expression of p65 significantly. Moreover, overexpression of NF-*κ*B-p65 markedly increased the MMP-7 expression that was decreased by knockdown of SLC12A5. The decreased migration and invasion by SLC12A5 knockdown in BUC cells was also rescued by overexpression of NF-*κ*B-p65.

In conclusion, our study showed that elevated expression of SLC12A5 increased the metastatic potential of BUC cells and that SLC12A5 promoted the migration and invasion of BUC cells through a process involving NF-*κ*B-dependent regulation of MMP-7 expression. Moreover, SLC12A5 is an independent prognostic factor for BUC patient survival. Targeting SLC12A5 could be a novel option to prevent BUC metastasis.

## Materials and methods

### Patients and specimens

Patient consent and approval from the Third Xiangya Hospital of Central South University Institutional Review Board were obtained for the use of these clinical materials for research purposes. Ten pairs of BUC tissue specimens and corresponding nontumorous specimens were obtained from patients with bladder BUC who underwent RC. All excised tissues were obtained within 1 h after surgery and were immediately placed in liquid nitrogen until further analysis.

IHC analyses were performed on 148 paraffin-embedded RC samples, which were histologically diagnosed as BUC at the Third Xiangya Hospital of Central South University and Sun Yat-sen University Cancer Center, between 2002 and 2012. The histological grade and stage were recorded according to the 2004 World Health Organization grading system and the sixth edition of the tumor node metastasis (TNM) classification system, respectively. The detail of patients' information is summarized in Table 1. The median follow-up period for this cohort of patients was 72 months (range, 36–156 months).

### IHC staining

The 148 paraffin-embedded BUC tissue specimens were cut into 2* μ*m sections and heated at 60 °C for 2 h. The sections were deparaffinized in xylene and rehydrated through descending alcohol concentrations. They were washed in PBS (pH 7.4) before being processed in EDTA (1 mM, pH 8.0) in a microwave oven at 100 °C for 15 min to expose antigenic sites, then cooled for 1 h at room temperature. Endogenous peroxidase activity was blocked using 0.3% hydrogen peroxide for 15 min at room temperature. The sections were incubated at 4 °C overnight in a humidified chamber with primary antibody against SLC12A5 antibody (1:100; Abcam, Cambridge, UK), MMP-7 (1:1000; Abcam) or PTEN (1:100; Santa Cruz Biotechnology, Santa Cruz, Delaware, USA). After washing in PBS, they were incubated with HRP-conjugated secondary antibody using an Envision Detection Kit, GK500705 (Gene Tech; Shanghai, China) for 30 min at room temperature. A negative control was prepared by replacing the primary antibody with PBS. Finally, the sections were incubated with 3,3-diaminobenzidine (DAB) and counterstained with hematoxylin and eosin (H&E) before being examined by light microscopy.

The resulting slides were assessed and scored according to the percentage of positive tumor cells as follows: the percentage of positive staining was defined as 0 (none positive staining cells), 1 (<20% of positive staining cells), 2 (20–50% of positive staining cells) or 3 (>50% of positive staining cells). The case with score 0, 1 or 2 was defined as low SLC12A5 expression, whereas the case with score 3 was regarded as high SLC12A5 expression. Previous scoring criteria were used for evaluation of the MMP-7 and PTEN IHC staining.^31, 32^

### Cells

The BUC cell lines T24 and BIU87 were maintained in RPMI 1640 medium (Invitrogen, Carlsbad, CA, USA) supplemented with 10% fetal bovine serum (HyClone, Logan, UT, USA), 100 U/ml penicillin and 100 *μ*g/ml streptomycin (Invitrogen) in humidified atmosphere containing 5% CO_2_ at 37 °C.

### Lentivirus packaging and transduction

The target sequences of SLC12A5 for constructing lentiviral short hairpin RNA were as follows: SLC12A5 ShRNA-1: 5′-GATCGGCAGCACAACACTGTGCTTGTTTCAAGAGAACAAGCACAGTGTTGTGCTGCTTTTTTC-3′; SLC12A5 ShRNA-2: 5′-GATCGGCGAGGTCATCACCATCTACTTTCAAGAGAAGTAGATGGTGATGACCTCGCTTTTTTC-3′. The MMP-7 and NF-*κ*B-p65 expression construct were generated by subcloning the PCR-amplified human MMP-7 and NF-*κ*B-p65 coding sequence into the pAd retroviral vector, respectively. Vector construction, lentivirus production and infection were performed as described previously.^33^

### RNA extraction and qRT-PCR

Total RNA was extracted from the freshly frozen paired tissue specimens using Trizol reagent (Invitrogen) according to the manufacturer's protocol. RNA concentration and purity were determined by absorbance at 260 nm using a NanoDrop ND-1000 spectrophotometer (NanoDrop Technologies; Houston, TX, USA). Reverse transcription (RT) was performed on 2 *μ*g of total RNA/sample using M-MLV reverse transcriptase (Promega; Madison, WI, USA) according to the manufacturer's instructions. Newly synthesized cDNA was amplified by qRT-PCR to enable the expression levels of SLC12A5 to be detected. The relative expressions of SLC12A5 were calculated by normalization against GAPDH. The primer sequences were as follows: SLC12A5, forward 5′-GCAGGAGCCATGTACATCCT-3′, reverse 5′-CCATGCAGGTGAGCACACA-3′ GAPDH, forward 5′-GGAGATTGTTGCCATCAACG-3′, reverse 5′-TTGGTGGTGCAGGATGCATT-3′. qRT-PCR was performed using SYBR Green Master Mix with an ABI 7900HT real-time PCR system (Life Technologies). The thermal profile consisted of an initial denaturation step at 95 °C for 10 min, followed by 40 cycles at 95 °C for 30 s and a final step at 60 °C for 1 min. The melting curve was determined at 95 °C for 15 s, 60 °C for 15 s and 72 °C for 15 s to confirm the specificities of the resulting products. The crossing threshold (CT) value of each sample was calculated during the exponential amplification phase using the instrument's software (SDS v.2.3). Data were analyzed using the comparative threshold cycle (2^−ΔΔCT^) method.

### Protein extraction and western blot

Freshly frozen tissue specimens and cell lines were suspended in ice-cold RIPA lysis buffer (Beyotime Technology; Shanghai, China). The samples were centrifuged at 12 000 g for 30 min at 4 °C and the supernatants were assayed to determine protein concentrations using a BCA Protein Assay Kit (Bio-Rad; Hercules, CA, USA). After quantification, 30 *μ*g protein from each sample was denatured before 12% SDS-PAGE electrophoreses and then transferred to PVDF membranes (Bio-Rad). Nonspecific binding was blocked by incubating the membranes in 8% nonfat milk for 1 h at room temperature. The membranes were then incubated overnight at 4 °C with anti-SLC12A5 antibody, anti-MMP-7, anti-PTEN, anti-BRMS1 (1:5000; Abcam), anti-KISS1 (1:1000; Abcam), CXCR2 (1:500; Abcam), anti-FGFR4 (1:1000; Abcam), FLT4 (1:500; Abcam), anti-MMP-3 (1:500; Cell Signaling Technology, Danvers, MA, USA), anti-MAGT5 (1:1000; Cell Signaling Technology), anti-TIMP-2 (1:200; Cell Signaling Technology), NF-*κ*B-p65 (1:500; Cell Signaling Technology) or anti-GAPDH (1:5000; Cell Signaling Technology) primary antibodies. After washing in phosphate-buffered saline-Tween (PBST), the membranes were incubated with Horseradish Peroxidase (HRP)-conjugated goat anti-rabbit antibody (1:5000; Calbiochem, Darmstadt, Germany) at room temperature for 1 h, then washed against in PBST. The immunoreactive proteins were visualized using enhanced chemiluminescence (ECL) detection reagent with an ECL kit (Cell Signaling Technology). The optical densities of the protein bands were measured using Quantity One software (Bio-Rad).

### Proliferation assay

For the cell proliferation assay, the growth rate of the infected cells was measured using an MTS cell proliferation assay. Briefly, the cells were plated in 96-well plates at a density of 1500 cells per well, and growth was evaluated using an MTS cell proliferation kit (Promega) according to the manufacturer's instructions.

### Cell migration and invasion assays

For the cell migration assay, 1 × 10^5^ cells were plated in the upper chamber (without Matrigel). For the cell invasion assay, 2 × 10^5^ cells were plated in the upper chamber, which was pre-coated with a thin layer of 0.5 mg/ml Matrigel (BD, Franklin Lakes, NJ, USA). For both assays, 600 *μ*l of RPMI 1640 medium supplemented with 10% FBS was added to the lower chamber, while 200 *μ*l of RPMI 1640 medium without FBS was placed in the upper chamber. The migration and invasion assays were performed after the cells were incubated at 37 °C for 48 h, respectively. The cells that remained in the upper chamber were removed with cotton-tipped swabs. The cells that had migrated or invaded to the lower chamber were then fixed in 75% ethanol for 30 min and stained with 0.5% crystal violet for 60 min. The migration and invasion efficiencies were evaluated by counting the stained cells on an inverted microscope (6 fields were randomly selected per membrane). Both assays were conducted 48 h after the T24 and BIU87 cells were infected. The cell migration was also assessed by measuring the movement of cells into a wound – a scraped, cellular area made by a 200 *μ*l pipette tube. Wounding closure was observed after 48 h.

### MMP-7 activity assay

The BUC cells were seeded in six-well plates and incubated at 37 °C. After 24 h, the medium was removed. Then, the cells were washed and incubated in serum-free medium for 48 h. The MMP-7 and activity in the media were detected using Fluorokine E Human MMP-7 Activity Assay kit (R&D Systems), according to the manufacturer's protocol.

### *In vivo* experiment

To produce experimental subcutaneous tumor growth, the BALB/c nude mice were randomly divided into four groups (T24-shSLC12A5 and T24-shCtrl; BIU87-shSLC12A5 and BIU87-shCtrl) consisting of four mice each. The mice were injected subcutaneously into the flank of mice with 5 × 10^6^ T24 and BIU87 cells. The tumor diameter was measured and the volume (width^2^ × length × 0.52) calculated every other day. The mice were humanely killed on day 45, and the tumors were dissected. To produce experimental metastasis, the BALB/c nude mice were randomly divided into four groups (T24-shSLC12A5 and T24-shCtrl; BIU87-shSLC12A5 and BIU87-shCtrl) consisting of six mice each. The mice were injected intravenously with 2.5 × 10^6^ T24 and BIU87 cells in 0.2 ml of PBS through the tail vein. After 2 months, the four groups of mice were killed, their lungs were resected and fixed in 10% buffered formalin for metastatic nodules counting. The number of metastatic nodules presented on the surface of each set of lungs was counted by visual inspection using a stereoscopic dissecting microscope. All the procedures are in accordance with the guidelines of the laboratory animal ethics committee of Central South University.

### Statistical analysis

Statistical analyses were carried out using SPSS v16.0 statistical software (SPSS, Chicago, IL, USA). Pearson's chi-square test (*χ*^2^) was used to determine the correlations between SLC12A5 expression levels and the clinical characteristics of patients with BUC. The Kaplan–Meier method and log-rank test were used to evaluate survival curves. The Cox proportional-hazard analysis was used for univariate and multivariate analyses to explore the effect of the clinicopathological variables and SLC12A5 expression on survival. Only the factors which were found to have statistically significant associations with survival based on a univariate analysis would be included in a multivariate Cox proportional hazards model to adjust for the effects of the covariates. A two-tailed unpaired Student's *t*-test was used to assess differences in cell proliferation rates, colony formation and cell migration and invasion between shSLC12A5- and shCtrl- transfected BUC cells. Statistical differences from at least three independent experiments were expressed as mean±standard deviation. A two-sided *P*-value <0.05 was considered to be statistically significant.

## Figures and Tables

**Table 1:** SLC12A5 expression and clinicopathological variables in bladder urothelial carcinoma

		SLC12A5 expression level		
Clinicopathological variables	Number of cases (*n*=148)	Low (%) (*n*=69)	High (%) (*n*=79)	*P*-value^a^
*Age (years)*				0.975
⩽60^b^	62	29 (46.8)	33 (53.2)	
>60	86	40 (46.5)	46 (53.5)	
				
*Gender*				0.742
Male	125	59 (47.2)	66 (52.8)	
Female	23	10 (43.5)	13 (56.5)	
				
*Tumor size (cm)*				0.781
⩽3.8^c^	84	40 (47.6)	44 (52.4)	
>3.8	64	29 (45.3)	35 (54.7)	
				
*Tumor multiplicity*				0.292
Unifocal	39	21 (53.8)	18 (46.2)	
Multifocal	109	48 (44.0)	61 (56.0)	
				
*Tumor grade*				0.13
Low	59	32 (54.2)	27 (45.8)	
High	89	37 (41.6)	52 (58.4)	
				
*pT status*				0.079
pT1	44	25 (56.8)	19 (43.2)	
pT2	52	26 (50.0)	26 (50.0)	
pT3/pT4	52	18 (34.6)	34 (65.4)	
				
*pN status*				**0.001**
pN−	123	65 (52.8)	58 (47.2)	
pN+	25	4 (16.0)	21 (84.0)	

a Chi-square test.

b Mean age.

c Mean size. Significant associations are shown in boldface in the *P*-value column (*P*-value <0.05)
