# Supplementary figures and images for: Solute carrier family 12 member 5 promotes tumor invasion/metastasis of bladder urothelial carcinoma by enhancing NF-κB/MMP-7 signaling pathway
Source: Cell Death Dis. 2017 Mar 23;8(3):e2691–. doi: 10.1038/cddis.2017.118 (PMC5386524; doi:10.1038/cddis.2017.118)

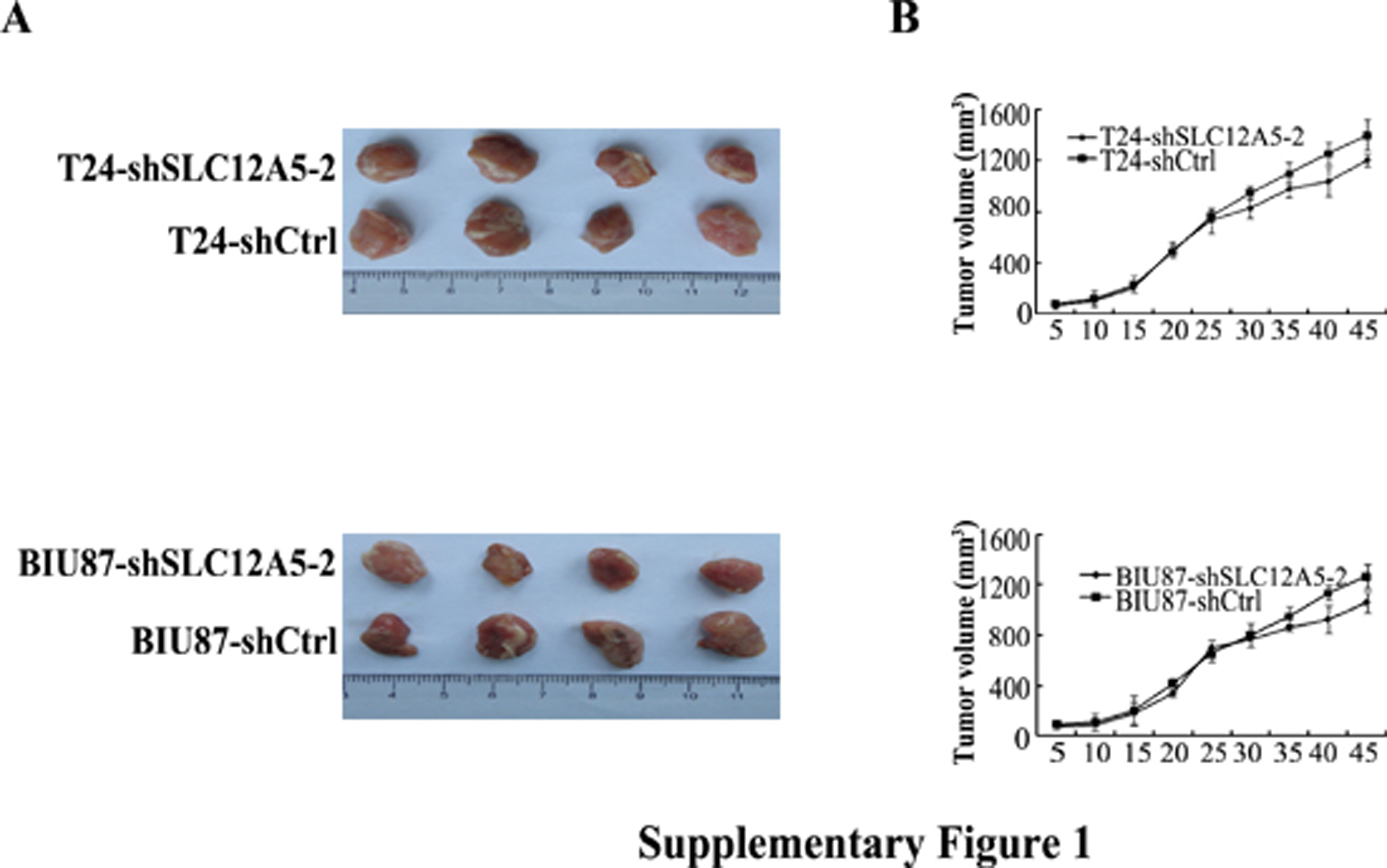

Supplement: Supplementary Figure 1 [file cddis2017118x1.tif]

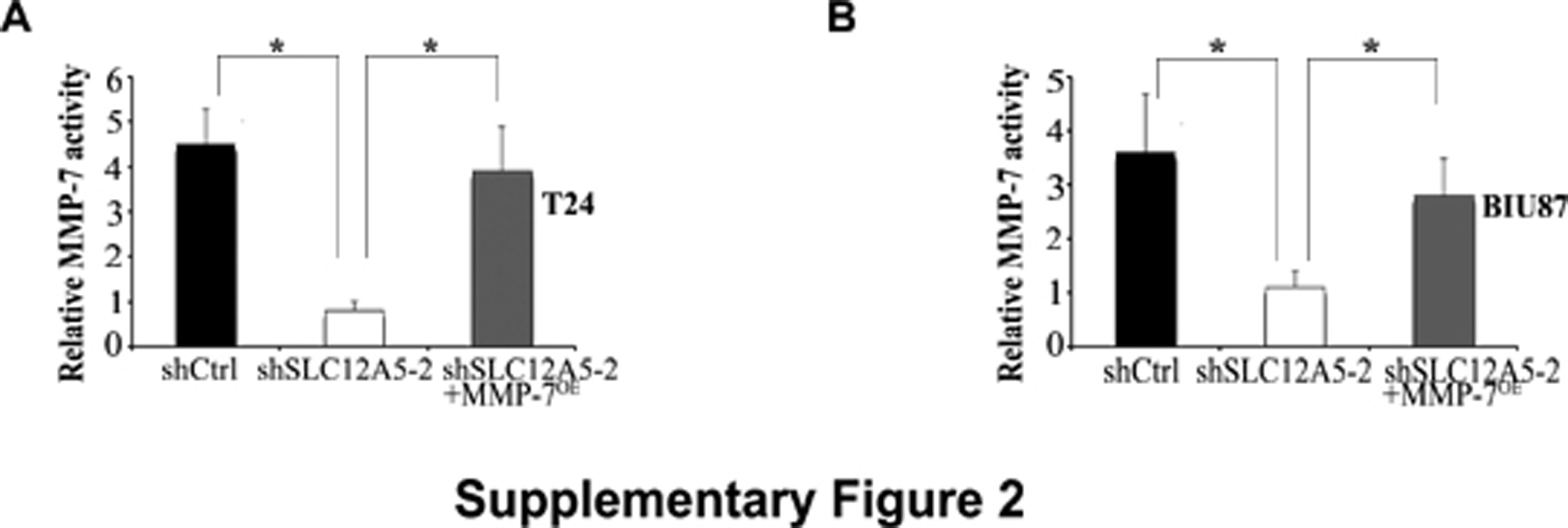

Supplement: Supplementary Figure 2 [file cddis2017118x2.tif]

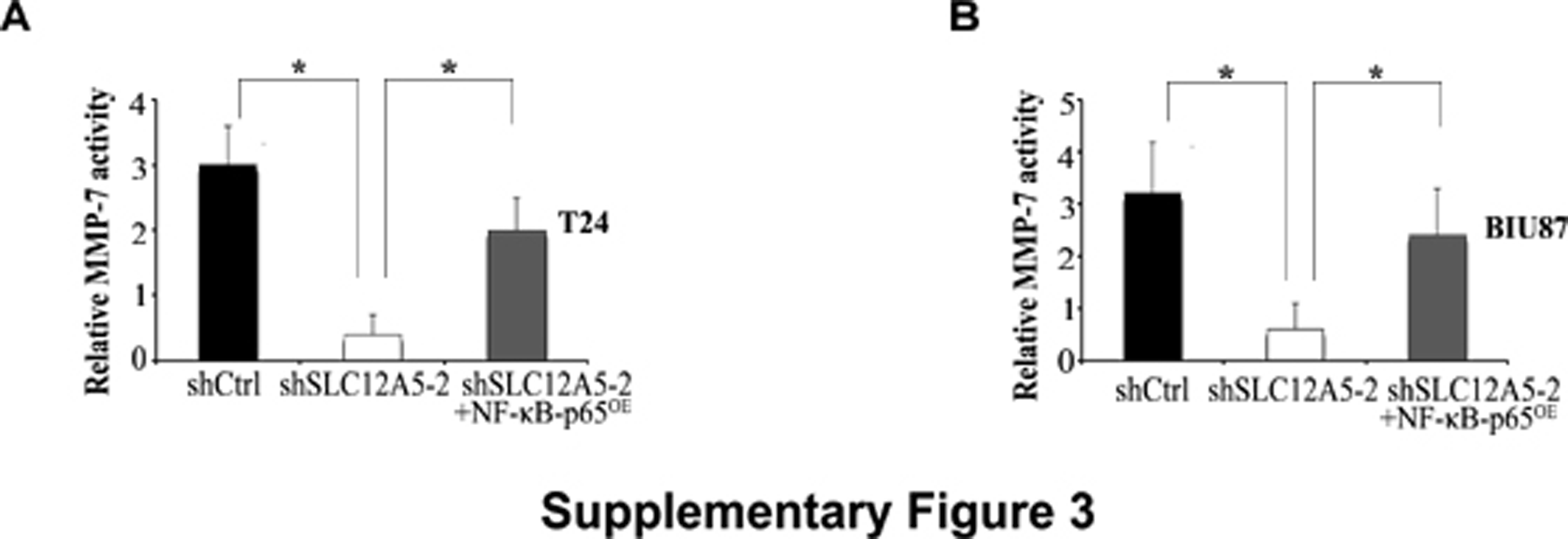

Supplement: Supplementary Figure 3 [file cddis2017118x3.tif]
