# Supplementary material for: Solute carrier family 12 member 5 promotes tumor invasion/metastasis of bladder urothelial carcinoma by enhancing NF-κB/MMP-7 signaling pathway
Source: Cell Death Dis. 2017 Mar 23;8(3):e2691–. doi: 10.1038/cddis.2017.118 (PMC5386524; doi:10.1038/cddis.2017.118)
Supplement: Supplementary Table 1 [file cddis2017118x4.docx]

| **Supplementary Table 1 Univariate cox proportional regression analysis for survival in bladder urothelial carcinom** | | | |  |
| --- | --- | --- | --- | --- |
| Variables | All cases | HR (95% CI) | *P* valueª |  |
| Age(years) |  |  | 0.39 |  |
| ≤60^b^ | 62 | 1 |  |  |
| ＞60 | 86 | 1.288 (0.723-2.296) |  |  |
| Gender |  |  | 0.934 |  |
| Male | 125 | 1 |  |  |
| Female | 23 | 0.967 (0.435-2.150) |  |  |
| Tumor size(cm) |  |  | 0.596 |  |
| ≤3.8^c^ | 84 | 1 |  |  |
| ＞3.8 | 64 | 1.166 (0.661-2.055) |  |  |
| Tumor multiplicity |  |  | 0.998 |  |
| Unifocal | 39 | 1 |  |  |
| Multifocal | 109 | 1.001 (0.545-1.837) |  |  |
| Tumor grade |  |  | **＜0.001** |  |
| Low | 59 | 1 |  |  |
| High | 89 | 4.092 (1.978-8.466) |  |  |
| pT status |  |  | **0.013** |  |
| pT1 | 44 | 1 |  |  |
| pT2 | 52 | 2.029 (0.847-4.860) |  |  |
| pT3/pT4 | 52 | 3.365 (1.454-7.789) |  |  |
| pN status |  |  | **＜0.001** |  |
| pN- | 123 | 1 |  |  |
| pN+ | 25 | 3.878 (2.110-7.127) |  |  |
| SLC12A5 |  |  | **＜0.001** |  |
| Low | 69 | 1 |  |  |
| High | 79 | 6.945 (3.238-14.896) |  |  |
| ^a^Chi-square test. ^b^mean age. ^c^mean size. HR: hazard ratio. CI: confidence interval. Significant associations are shown in bold face in the *p*-value column (*p*-value <0.05). | | | | |
