# Supplementary material for: Solute carrier family 12 member 5 promotes tumor invasion/metastasis of bladder urothelial carcinoma by enhancing NF-κB/MMP-7 signaling pathway
Source: Cell Death Dis. 2017 Mar 23;8(3):e2691–. doi: 10.1038/cddis.2017.118 (PMC5386524; doi:10.1038/cddis.2017.118)
Supplement: Supplementary Table 2 [file cddis2017118x5.docx]

| **Supplementary Table 2 Multivariate cox proportional regression analysis for survival in bladder urothelial carcinom** | | | |
| --- | --- | --- | --- |
| Variables | Hazards ratio | 95% CI^a^ | *P* value |
| Tumor grade ( low vs. high) | 3.508 | 1.632-7.538 | **0.001** |
| pT status ( pT1 vs. pT2 vs. pT3/pT4) | 1.491 | 1.013-2.193 | **0.043** |
| pN status ( pN- vs. pN+) | 2.066 | 1.109-3.850 | **0.022** |
| SLC12A5 (low vs. high) | 2.323 | 1.820–2.966 | **＜0.001** |
| ^a^CI: confidence interval. Significant associations are shown in bold face in the *p*-value column (*p*-value <0.05). | | | |
