# Supplementary material for: Solute carrier family 12 member 5 promotes tumor invasion/metastasis of bladder urothelial carcinoma by enhancing NF-κB/MMP-7 signaling pathway
Source: Cell Death Dis. 2017 Mar 23;8(3):e2691–. doi: 10.1038/cddis.2017.118 (PMC5386524; doi:10.1038/cddis.2017.118)
Supplement: Supplementary Table 3 [file cddis2017118x6.docx]

| **Supplementary Table 3 List of genes differentially expressed in bladder urothelial carcinoma T24 cells afterSLC12A5 knockdown using a Human Tumor Metastasis Real-time PCR Array** | | | |
| --- | --- | --- | --- |
| Gene | Fold change | Location | Function |
| Upregulated genes |  |  |  |
| B2M | 1.05 | 15q21-q22.2 | immune response, MHC I receptor |
| APC | 1.67 | 5q21-q22 | cell adhesion, inhibits proliferation |
| BRMS1 | 3.48 | 11q13-q13.2 | inhibits metastasis and tumor growth |
| CD44 | 1.39 | 11p13 | cell adhesion and stroma attachment |
| CDH1 | 1.82 | 16q22.1 | inhibits tumor metastasis |
| CDKN2A | 1.2 | 9p21 | negative regulation of cell cycle |
| CTBP1 | 1.34 | 4p16 | inhibits cell proliferation |
| CTNNA1 | 1.33 | 5q31 | participates in cell adhesion |
| CTSL1 | 1.02 | 9q21-q22 | protein hydrolysate |
| CTSK | 1.35 | 1q21 | protein hydrolysate |
| CXCR4 | 1.36 | 2q21 | signal transduction, promotes invasion |
| DENR | 1.08 | 12q24.31 | promotes proliferation |
| EWSR1 | 1.26 | 22q12.2 | transcription factor, promotes oncogenesis |
| FN1 | 1.69 | 2q34 | participates in cell adhesion |
| FAT1 | 1.27 | 4q35 | participates in cell adhesion |
| HPSE | 1.37 | 4q21.3 | hydrolyses protein |
| HRAS | 1.51 | 11p15.5 | promotes proliferation |
| ITGB3 | 1.23 | 17q21.32 | participates in cell adhesion |
| IL1B | 1.5 | 2q14 | inhibits or promotes proliferation |
| IL18 | 1.68 | 11q22.2-q22.3 | promotes cell proliferation |
| IGF1 | 1.35 | 12q22-q23 | promotes proliferation |
| KRAS | 1.78 | 12p12.1 | cell signal transduction, proliferation |
| KISS1 | 2.45 | 1q32 | suppresses metastasis |
| MYCL1 | 1.02 | 1p34.2 | transcription factor, promotes proliferation |
| MYC | 1.09 | 8q24.12-q24.13 | promotes proliferation |
| MMP9 | 1.07 | 20q11.2-q13.1 | decomposes, protein hydrolysate  and promotes metastasis |
| MMP13 | 1.25 | 11q22.3 | decomposes, protein hydrolysate  and promotes metastasis |
| NR4A3 | 1.9 | 9q22 | transcription factor, promotes proliferation |
| PTEN | 6.67 | 10q23.3 | inhibits proliferation and metastasis |
| RB1 | 1.32 | 13q14.2 | negative regulation of cell reproduction |
| SRC | 1.6 | 20q12-q13 | promotes proliferation |
| TNFSF10 | 1.14 | 3q26 | induces apoptosis, inhibits proliferation |
| TIMP2 | 1.25 | 17q25 | inhibits metastasis |
| TIMP3 | 1.34 | 22q12.3 | induces apoptosis, inhibits metastasis |
| TIMP4 | 1.09 | 3p25 | inhibits metastasis |
| TCF20 | 1.26 | 22q13.3 | transcription factor |
| Downregulated genes |  |  |  |
| ACTB | -1.23 | 7p15-p12 | ORM cytoskeleton |
| CDH6 | -1.01 | 5p15.1-p14 | osteosis, cell adhesion |
| CDH11 | -1.47 | 16q22.1 | involves in the metastatic process |
| CHD4 | -1.35 | 12p13 | chromatin assemble and modification |
| CD82 | -1.98 | 11p11.2 | metastasis suppressor |
| COL4A2 | -1.29 | 13q34 | the component of extracellular matrix |
| CST7 | -1.41 | 20p11.21 | inhibits cysteine proteinase |
| CXCR2 | -9.05 | 2q35 | signal transduction, promotes invasion |
| CXCL12 | -1.48 | 10q11.1 | participates in cell adhesion |
| EPHB2 | -1.7 | 1p36.1-p35 | signal transduction, promotes invasion |
| ETV4 | -1.23 | 17q21 | transcription factor, promotes proliferation |
| FGFR4 | -2.76 | 5q35.1 | promotes invasion |
| FXYD5 | -1.64 | 19q12-q13.1 | negative regulation of cell adhesion |
| FLT4 | -3.32 | 5q34-q35 | promotes tumor metastasis |
| GAPDH | -1.17 | 12p13 | glycometabolism |
| GNRH1 | -1.26 | 8p21-p11.2 | inhibits cell proliferation |
| HGF | -1.42 | 7q21.1 | participates in proteolysis,  promotes proliferation |
| HPRT1 | -1.26 | Xq26.1 | nucleotide metabolism |
| HTATIP2 | -1.67 | 11q13 | positively regulates transcription |
| ITGA7 | -1.59 | 12q13 | participates in cell adhesion |
| KISS1R | -1.03 | 19p13.3 | suppresses metastasis |
| MCAM | -1.25 | 11q23.3 | participates in cell adhesion |
| MTA1 | -1.76 | 14q32.3 | promotes metastasis |
| MMP2 | -1.89 | 16q13-q21 | decomposes, protein hydrolysate  and promotes metastasis |
| MMP3 | -2.56 | 11q22.3 | decomposes, protein hydrolysate  and promotes metastasis |
| MMP7 | -6.89 | 11q21-q22 | decomposes, protein hydrolysate  and promotes metastasis |
| MMP10 | -1.38 | 11q22.3 | protein hydrolysate, promotes metastasis |
| MMP11 | -1.87 | 22q11.23 | decomposes, protein hydrolysate  and promotes metastasis |
| MTSS1 | -1.23 | 8p22 | inhibits metastasis and proliferation |
| MGAT5 | -4.26 | 2q21 | promotes metastasis |
| METAP2 | -1.43 | 12q22 | protein hydrolysate and modification |
| MDM2 | -1.28 | 12q14.3-q15 | negative regulation of cell proliferation |
| MET | -1.72 | 7q31 | proto-oncogene, promotes cell proliferation |
| NME4 | -1.32 | 16p13.3 | inhibits proliferation |
| NME1 | -1.65 | 17q21.33 | suppresses metastasis |
| NF2 | -1.05 | 22q12.2 | inhibits proliferation |
| PNN | -1.92 | 14q21.1 | inhibits proliferation |
| PLAUR | -1.23 | 19q13 | activator of plasminogen |
| RPSA | -1.84 | 3p22.2 | cell adhesion and protein biosynthesis |
| RPLPO | -1.52 | 12q24.2 | protein biosynthesis |
| RORB | -1.2 | 9q22 | participates in regulate of transcription |
| SET | -1.51 | 9q34 | inhibits histone acetylation |
| SYK | -1.19 | 9q22 | promotes proliferation |
| SMAD4 | -1.03 | 18q21.1 | cell signal transduction |
| SMAD2 | -1.47 | 18q21.1 | cell signal transduction |
| SERPINE1 | -1.27 | 7q22.1 | negative regulation fibrinolysis |
| SSTR2 | -1.14 | 17q24 | inhibits proliferation |
| TSHR | -1.76 | 14q31 | promotes proliferation |
| TRPM1 | -1.82 | 15q13-q14 | calcium channels |
| TP53 | -1.69 | 17p13.1 | induces apoptosis and cell differentiation,  inhibits proliferation |
| TGFB1 | -1.04 | 19q13.1 | inhibits or promotes proliferation,  promotes metastasis |
| VEGFA | -1.69 | 6p12 | promotes proliferation, metastasis;  inhibits apoptosis |
