# Supplementary material for: Solute carrier family 12 member 5 promotes tumor invasion/metastasis of bladder urothelial carcinoma by enhancing NF-κB/MMP-7 signaling pathway
Source: Cell Death Dis. 2017 Mar 23;8(3):e2691–. doi: 10.1038/cddis.2017.118 (PMC5386524; doi:10.1038/cddis.2017.118)
Supplement: Supplementary Table 4 [file cddis2017118x7.docx]

| **Supplementary Table 4 Association between expression of SLC12A5 and PTEN and MMP-7 in bladder urothelial carcinom** | | | | |
| --- | --- | --- | --- | --- |
|  |  | SLC12A5 | |  |
| Variables | Cases | Low(%) | High(%) | *P* value^a^ |
| PTEN |  | 69 | 79 |  |
| Negative | 113 | 53(46.9%) | 60(53.1%) | 0.902 |
| Positive | 35 | 16(45.7%) | 19(54.3%) |  |
| MMP-7 |  |  |  |  |
| Negative | 56 | 42(75.0%) | 14(25.0%) | **＜0.001** |
| Positive | 92 | 27(29.3%) | 65(70.7%) |  |
| ^a^Fisher’s exact test. Significant associations are shown in bold face in the *p*-value column (*p*-value <0.05). | | | | |
